# Supplementary material for: Alcohol Consumption and Risk of Rheumatoid Arthritis among Chinese Adults: A Prospective Study
Source: Nutrients. 2021 Jun 29;13(7):2231. doi: 10.3390/nu13072231 (PMC8308382; doi:10.3390/nu13072231)
Supplement: Supplementary file 1 [file nutrients-13-02231-s001.zip › nutrients-1218207-supplementary.pdf]

Supplementary Table S1. Adjusted hazard ratio and 95% confidence interval for risk of rheumatoid arthritis (RA) by baseline wine, beer, or liquor consumption group[a]

	Wine, Beer, or Liquor		
	**Never or past**	**Light or Moderate**	**Heavy**
Wine and Beer			
# of case / population	82/81532	4/5532	1/49
Incidence rate (/10000 person-years)	1.25	0.83	23.78
Multivariate-adjusted hazard ratio[b]	1.00 (Ref.)	0.89 (0.32, 2.49)	-
Liqour			
# of case / population	67/66541	8/9795	12/10782
Incidence rate (/10000 person-years)	1.25	0.99	1.36
Multivariate-adjusted hazard ratio[b]	1.00 (Ref.)	1.24 (0.55, 2.79)	1.69 (0.80, 3.58)

[a] Alcohol consumption is grouped into never or past, light to moderate (women: 0–1.0 servings/d; men: 0–2.0 servings/d), and heavy (women: >1.0 serving/d; men: >2 servings/d) for wine and beer combined, and liquor separately

[b] Adjusted for sex, age, body mass index (< 18.5 kg/m2, 18.5- 23 kg/m2, 23-27.5 kg/m2, > 27.5 kg/m2), and smoking (never, past, current)

Supplementary Table S2. Adjusted hazard ratio and 95% confidence interval for risk of rheumatoid arthritis (RA) subtypes by baseline alcohol consumption group[a]

	Alcohol		
	Never or past	Light or Moderate	Heavy
**Seropositive[b] RA**			
# of case / population	51/61161	12/15113	10/10844
Incidence rate (/10000 person-years)	1.05	0.94	1.13
Multivariate-adjusted hazard ratio[c]	1.00 (Ref.)	1.46 (0.70, 3.04)	1.64 (0.71, 3.82)
**Seronegative[d] RA**			
# of case / population	11/61161	0/15113	3/10844
Incidence rate (/10000 person-years)	0.23	-	0.34
Multivariate-adjusted hazard ratio[b]	1.00 (Ref.)	-	-
**RF Positive[e] RA**			
# of case / population	50/61161	11/15113	10/10844
Incidence rate (/10000 person-years)	1.03	0.86	1.13
Multivariate-adjusted hazard ratio[b]	1.00 (Ref.)	1.37 (0.65, 2.92)	1.92 (0.84, 4.37)
**RF Negative[f] RA**			
# of case / population	12/61161	1/15113	2/10844
Incidence rate (/10000 person-years)	0.25	0.08	0.23
Multivariate-adjusted hazard ratio[b]	1.00 (Ref.)	0.64 (0.07, 5.64)	3.84 (0.75, 19.69)
**a-CCP Positive[g] RA**			
# of case / population	37/61161	1/15113	4/10844
Incidence rate (/10000 person-years)	0.76	0.08	0.45
Multivariate-adjusted hazard ratio[b]	1.00 (Ref.)	1.61 (0.76, 3.42)	1.41 (0.56, 3.51)
**a-CCP Negative[h] RA**			
# of case / population	18/61161	0/15113	5/10844
Incidence rate (/10000 person-years)	0.37	-	0.56
Multivariate-adjusted hazard ratio[b]	1.00 (Ref.)	-	-

[a] Alcohol consumption is grouped into never or past, light to moderate (women: 0–1.0 servings/d; men: 0–2.0 servings/d), and heavy (women: >1.0 serving/d; men: >2 servings/d)
[b] Positive for rheumatoid factor (RF), anti-citrullinated protein antibodies (a-CCP), or both
[c] Adjusted for sex, age, body mass index (< 18.5 kg/m2, 18.5- 23 kg/m2, 23-27.5 kg/m2, > 27.5 kg/m2), and smoking (never, past, current)
[d] Negative for RF and a-CCP
[e] Positive for RF, regardless of a-CCP status
[f] Negative for RF, regardless of a-CCP status
[g] Positive for a-CCP, regardless of RF status
[h] Negative for a-CCP, regardless of RF status
